# Thermo-Responsive Sol-Gel-Based Nano-Carriers Containing Terbinafine HCl: Formulation, In Vitro and Ex Vivo Characterization, and Antifungal Activity

**DOI:** 10.3390/gels9100830

**Published:** 2023-10-20

**Authors:** Maryam Bajwa, Naila Tabassam, Huma Hameed, Ali Irfan, Muhammad Zaman, Mahtab Ahmad Khan, Gamal A. Shazly, Tooba Mehboob, Tehseen Riaz, Yousef A. Bin Jardan

**Affiliations:** 1 Faculty of Pharmaceutical Sciences, University of Central Punjab, Lahore 54000, Pakistan; 2 Department of Chemistry, Government College University Faisalabad, Faisalabad 38000, Pakistan; raialiirfan@gmail.com; 3 Institute of Clinical and Experimental Pharmacology and Toxicology, University of Lubeck, 23566 Lubeck, Germany; 4 Department of Pharmaceutics, College of Pharmacy, King Saud University, Riyadh 11451, Saudi Arabia

**Keywords:** sol-gel based nanocarriers, terbinafine, poloxamer-407, poloxamer-188, nail infection, gelation temperature, zeta potential

## Abstract

The current research aims to create a sol-gel-based nanocarrier containing terbinafine formulated for transdermal delivery of the drug into the skin. Sol-gel-based nanocarriers were prepared via the cold method using poloxamer-188, poloxamer-407, and distilled water. The prepared formulation was examined for pH, gelation temperature, Fourier transform infrared spectrophotometer (FTIR) analysis, thermal stability analysis, X-ray diffraction (XRD), scanning electron microscopy (SEM), particle size analysis, zeta potential, and anti-microbial activity. The in-vitro drug release study of F1 was found to be 94%, which showed greater drug release as compared to F2 and F3. The pH of the formulation was found to be within the range applicable to the skin. The gelation temperature was detected at 28 °C. The SEM images of formulations have spotted various particles well-segregated from each other. Analysis of formulations showed a mean globule size diameter of 428 nm, zeta potential values of 0.04 mV, refractive index (1.329), and viscosity (5.94 cP). FTIR analysis confirmed various functional groups’ presence in the prepared formulation. Thermal analysis has confirmed the stability of the drug within the prepared formulation. The growth of inhibition was found to be 79.2% in 60 min, which revealed that the prepared formulation has shown good permeation from the membrane. Hence, the sol-gel-based nanocarrier formulation of terbinafine was successfully developed and evaluated.

## 1. Introduction

Onychomycosis is the most common fungal infection of nails and is responsible for approximately 50% of all nail diseases. Its prevalence in the general population ranges from 2–39% [1]. The disease is caused by dermatophytes, non-dermatophytes, and yeast [2]. It makes the nail fragile, thick, brittle, and discolored. It has been discovered that a fungus infection damages the toenail more than any other fingernail [1,2,3,4,5,6]. Onychomycosis can occur in children, but it is more common in adults and the elderly, indicating a correlation with age [4]. Despite proper diagnosis and the availability of antifungal treatments, onychomycosis is associated with treatment failure and increased reoccurrence rates [5]. Oral antifungals are typically the most popular form of treatment but have drawbacks like hepatotoxicity, adverse reactions, and drug-drug interactions, making them ineffective for most patients [6]. The topical formulation also faced challenges of penetration and being absorbed through the nail plate [7].

Terbinafine is a broad-spectrum allylamine antifungal available in oral and topical formulations [8]. It is one of the typical therapeutic regimens for onychomycosis. In the literature, it was already reported that terbinafine has a broad-spectrum fungicidal effect by inhibiting the enzyme squalene monooxygenase, which is involved in the synthesis of sterol in fungi. This inhibits fungal sterol biosynthesis by decreasing ergosterol levels. On average, this drug results in a 70–79% recovery rate, with complete recovery ranging from 38–59% [9]. After oral administration, terbinafine HCl undergoes the first-pass effect and is extensively bound to the plasma proteins; therefore, a high dose is recommended [10]. This topical route is preferred for its effective antimycotic activity. Furthermore, its topical administration also minimizes its systemic toxicity [11]. Terbinafine HCl belongs to BCS class II and has poor solubility and high permeability. To improve the solubility of such drugs, various techniques or methods are developed, like nanotechnology, solid dispersion techniques, particle size reduction, etc. [12]. Over the past few years, in situ gel systems have emerged as a novel approach for sustained and controlled drug release because of their particular property of transforming sol into gel [13].

Sol-gel-based nanocarriers are in a liquid state under specific conditions of temperature, pH, and ionic strength, and this state is subsequently transferred into a gel as the conditions are changed [14]. The sol-gel-based nanocarriers method stands as an appealing approach for formulating products due to its capacity to uphold product purity while also providing a large surface area [9]. Meanwhile, this approach is cost-effective, simple, and rapid. It does not require the use of an increased temperature or the pretreatment of biomaterial surfaces. It is a versatile process that allows the incorporation of various functional molecules [15]. Moreover, a sol is defined as a stable liquid suspension of colloidal particles (nanoparticles). These particles might be crystalline or amorphous, and their substructures can be dense, porous, or polymeric [16].

Formulation of sol-gel-based nanocarriers involves various mechanisms, including a temperature- or pH-dependent system, solvent exchange and swelling techniques, or chemical transformation to evolve a gel matrix of distinct solid and liquid phases [17,18]. Gel prepared by this technique is capable of casting. Thus, these gels can be molded and dried to produce membranes or filters too [19].

Previously used polymers for gels, such as starch, gelatin, chitosan, etc., as carriers in drug delivery were fraught with many formulation problems such as purity, instability, irreproducibility, changes in aesthetics on storage, uncontrollable formulation characteristics, etc., even though they are biocompatible and biodegradable. Hence, a wide spectrum of new designer polymers, such as poloxamers and polymeric hydrogel-based nanocarriers synthesized using appropriately chosen monomers, can solve many of the previously reported problems [1]. Secondly, poloxamers are thermoresponsive polymers, which are ABA triblock copolymers with A and B blocks being based on EG and propylene glycol (PG), respectively. While EG is highly hydrophilic and shows a thermoresponse at high temperatures, PG is thermoresponsive below, respectively. That is the novelty of these polymers, which provide targeted and controlled effects at the sight of rapid permeation and also enhance the stability of formulations because of their sol-gel and gel-sol nature [17].

**Figure 1:**
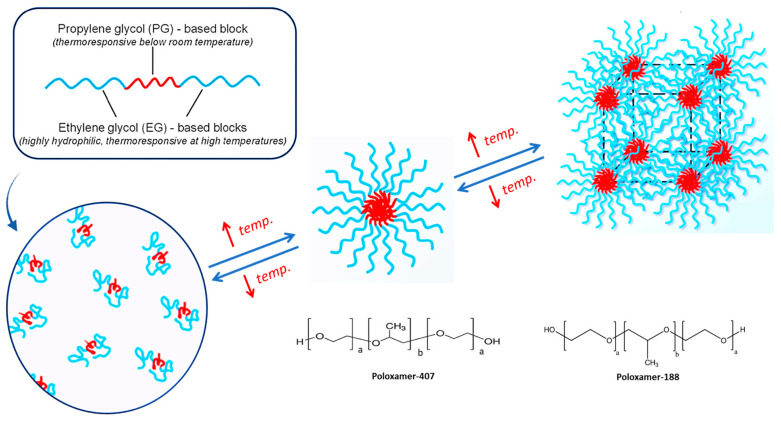
Schematic representation of thermoresponsive polymer (Poloxamer) in micelle formation; a,b,a triblock copolymers with “a” and “b” blocks being based on ethylene glycol (EG) and propylene glycol (PG), respectively.

Poloxamers 407 and 188 are two commonly used types of poloxamers. They self-assemble into micelles, have thermal reversibility, and are biocompatible without causing skin irritation. Poloxamer 407 has the ability to control the release system, while 108 can modulate the phase transition interval of 407. We utilize the thermoreversible nature of poloxamers 407 and 108 to develop a transdermal sol-gel [18]. The presence of both hydrophobic and hydrophilic groups in this polymer allows terbinafine to be soluble at skin pH. A schematic representation is shown below in Figure 1.

In the current formulation, sol-gel-based nanocarriers were prepared by the “cold method”. This method utilized polymers, specifically thermoresponsive poloxamers of grades 188 and 407 [20]. The commercially available poloxamer is used as an emulsifying, gelling agent, or solubility enhancer. It is water-soluble, and its concentrated solution forms thermoreversible gels. These gels are used as carriers for poorly soluble drugs [21]. The objective of this study was to develop terbinafine-HCl-containing sol-gel-based nanocarriers for better treatment of onychomycosis, which are further characterized for in vitro and permeation studies as well [22]. Terbinafine-containing sol-gel-based nanocarriers are available as sol, which transforms into gel upon contact with body temperature after being applied to the infected nail.

## 2. Results

### 2.1. Formulation Optimization Data Analysis

Table 1 displays the coded values of two components based on preliminary investigations.

**Figure 2:**
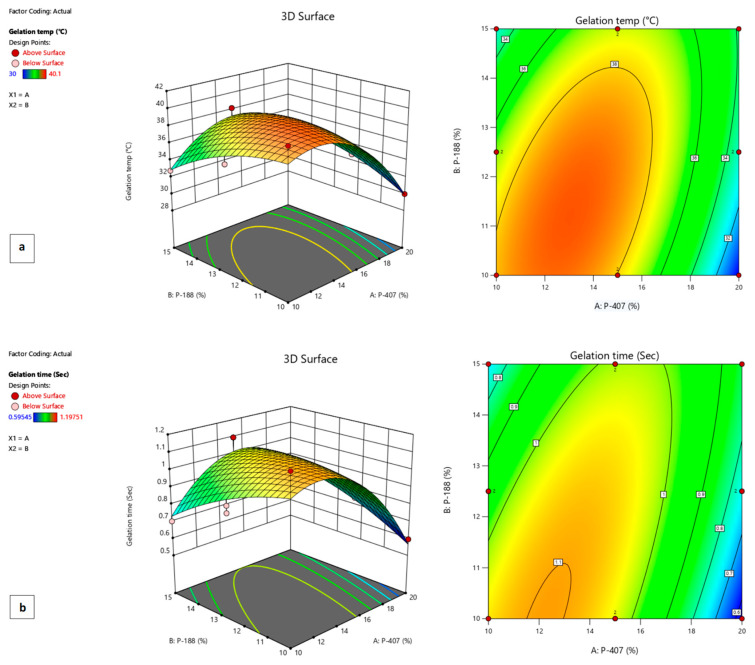
Response surface plots showing (**a**) the effects of poloxamers 407 and 188 on gelation temperature and (**b**) the effects of poloxamers 407 and 188 on gelation time of F1 formulation.

The *p*-value was less than 0.05 and showed that the model terms were significant for gelation temperature and gelling time. The model analysis also described the inverse relationship between gelation temperature and gelling time with poloxamer 407 and a direct relationship with poloxamer 188. In addition, as the concentration of poloxamer 407 increased, gelling time decreased while increasing with an increased concentration of poloxamer 188. Further, the analysis of both responses showed that thermoreversible polymers were active in the formulation. The effects of poloxamers 407 and 188 on gelation temperature and gelation time are also illustrated in Figure 2, respectively.

The value of R^2^ revealed the positive correlation coefficient between the models. The R^2^ value for gelation temperature was 0.86 and for gelling time was 0.78, as shown in Table 2.

The effect of polymers used were seen on a gelation temperature and gelling time as shown in Figure 2. The use of the polynomial equation is shown below in Equations (1) and (2).

Gelation temperature = 38.82 β_0_ − 1.84 X_1_ − 0.36X^2^ + 2.33X_1_X_2_ − 3.87X_1_^2^ − 1.22X_2_^2^ (1)

Gelling time = 67 β_0_ − 0.09 X_1_+ 0.0125X^2^ + 0.75X_1_X_2_ − 0.225X_1_^2^ − 0.047X_2_^2^ (2)

### 2.2. Clarity

**Figure 3:**
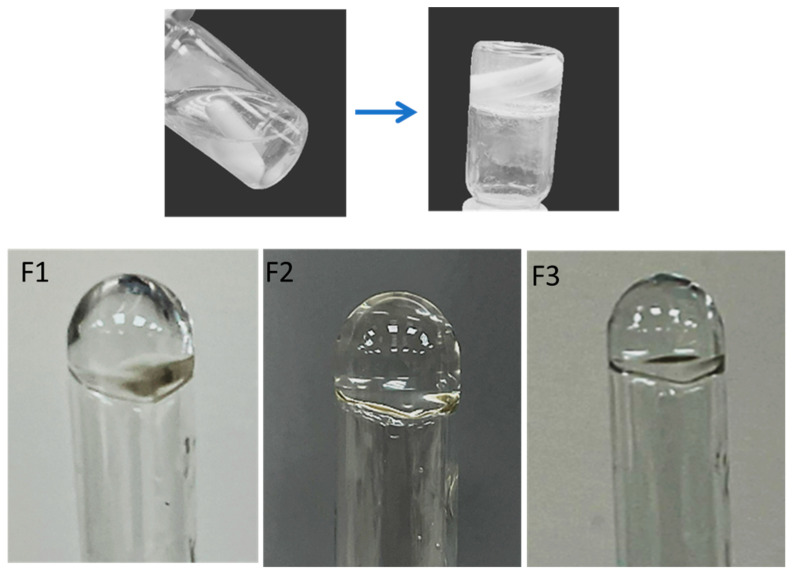
Visual inspection of the formulation of F1–F3 formulations.

All three formulations were checked visually for clarity against a dark and white background. These were clear and had no particles, as well as being free-flowing at room temperature, as shown in Figure 3.

### 2.3. Composition and pH of F1–F3 Formulations

pH readings of the optimized formulations and their composition are mentioned in Table 3. The normal physiological pH of skin ranges from 4.2–6.4 [22]. The pH of the sol-gel-based nanocarrier formulation was found to be within the range that makes it a non-irritant.

### 2.4. Viscosity

The viscometer BDV-8S was operated to measure the viscosity at 60 rpm with spindle 3. The results were within the range shown in Table 4.

### 2.5. Gelation Temperature

The formulation was poured into a clean glass vial. Then, a magnetic stirrer with a heating element is used to position this vial. The temperature is progressively raised while the formulation is agitated at the slowest possible pace. The temperature at which the magnetic bead stops rotating is identified as the gelation temperature [23]. The gelation temperature of the formulation is mentioned in Table 5.

### 2.6. Fourier Transform Infrared Spectrophotometer (FTIR)

**Figure 4:**
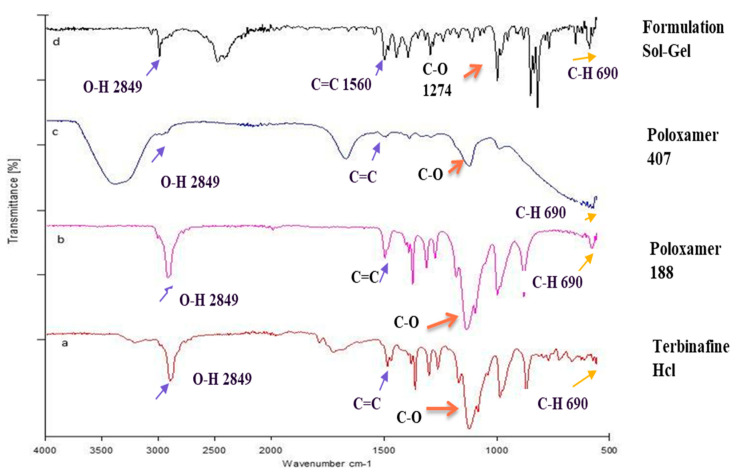
FTIR spectrum: a—drug; b—poloxamer 188; c—poloxamer 407; d—formulation.

**Figure 5:**
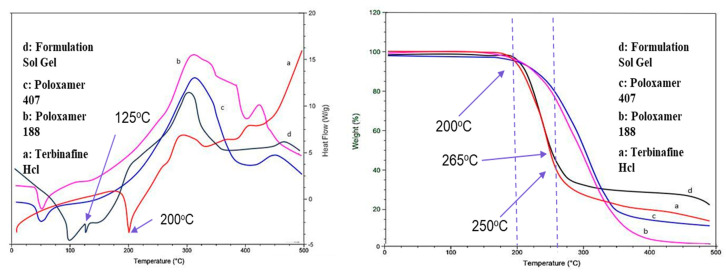
DSC/TGA analysis of the optimized formulation: a—drug; b—poloxamer 188; c—poloxamer 407; d—formulation.

FTIR has been used effectively for finding the different bonding arrangements and crystal packings in various organic compounds. FTIR analysis was performed on all the excipients and the drug. Various peaks emerged, indicating different functional groups. The FTIR spectrum of poloxamer 407 (depicted in Figure 4c) was observed across wavelengths ranging from 500 cm^−1^ to 4000 cm^−1^. Noteworthy absorption peaks in the IR spectrum of poloxamer 407 appeared at 2891 cm^−1^ (C-H aliphatic stretching), 1343 cm^−1^ (in-plane O-H bending), and 1111 cm^−1^ (C-O stretching). The FTIR spectrum of poloxamer 188 (Figure 5b) was observed between the wavelengths of 500 cm^−1^ and 4000 cm^−1^. The FTIR spectrum of poloxamer 188 showed the characteristic peak at 2885 cm^−1^ (C-H stretch aliphatic) and at 1344.1 cm^−1^ (in-plane O-H bending), comparable to the previously reported data, which also showed the band at 1099.2 cm^−1^ representative of C-O stretching and is exactly identical to the earlier reported value by Sharma et al. [24]. Terbinafine displayed a distinctive functional group peak at wavenumbers of 3012 cm^−1^ (alkenyl C-H stretch), 2921 cm^−1^ (alkyl C-H stretch), and 2223 cm^−1^ (alkenyl C≡C stretch). The FTIR spectrum of the prepared formulation (Figure 4d) was observed between the wavelengths of 500 cm^−1^ and 4000 cm^−1^. Only a shift in the frequencies of the bands was observed, which indicates physical interaction between the excipients and drug and no chemical interaction.

### 2.7. Thermal Stability Analysis (DSC/TGA)

The stability of an optimized formulation (F1), among various considerations, relies on the harmony between the drug and the excipients. Potential interactions between the drug and the employed polymer were examined by studying the DSC thermograms of the formulation. Differential Scanning Calorimetry (DSC) profiles were generated for both Terbinafine HCl and the physical blend within the gel, as mentioned in Figure 5. A sharp endothermic fusion peak at 200 °C represents the melting point of Terbinafine HCl, and a decrease in temperature to 125 °C is an indicator of enhanced drug solubility.

### 2.8. X-ray Diffraction (XRD)

**Figure 6:**
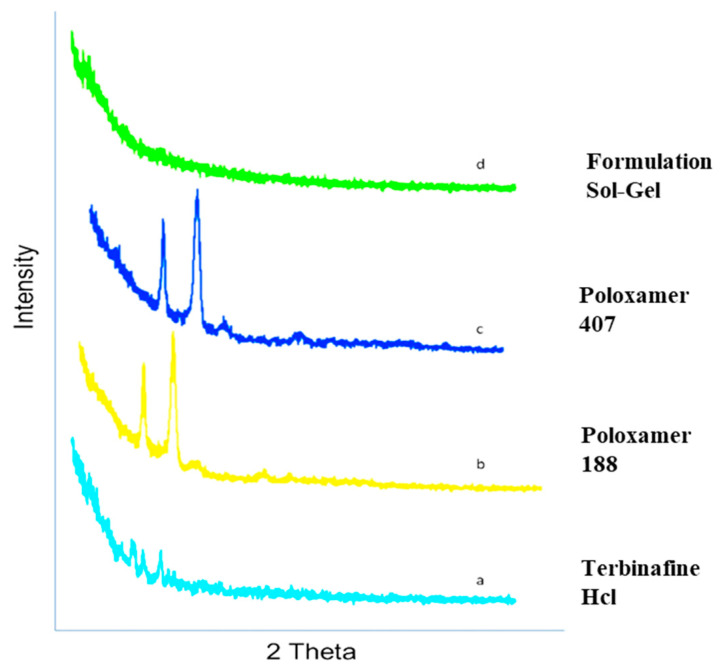
XRD pattern of optimized formulation: a—drug; b—poloxamer 188; c—poloxamer 407; d—formulation.

Figure 6 represents the XRD pattern of terbinafine sol-gel-based nanocarriers. X-ray diffraction (XRD) is a commonly employed technique for assessing the crystalline characteristics of the prepared formulation. Typically, the XRD patterns of the sol-gel-based nanocarrier formulation (F1) sample exhibit strong and well-defined peaks. The peak of the pure drug terbinafine HCl was clearly observed. The characteristic peaks of Terbinafine HCl appeared at a diffraction angle of 77.24 and a maximum intensity of 324. The reduction in diffraction peaks shifts, suggesting that the drug in the formulation is available in the amorphous form, which indicates the enhanced solubility of the drug.

### 2.9. Scanning Electron Microscopy (SEM)

**Figure 7:**
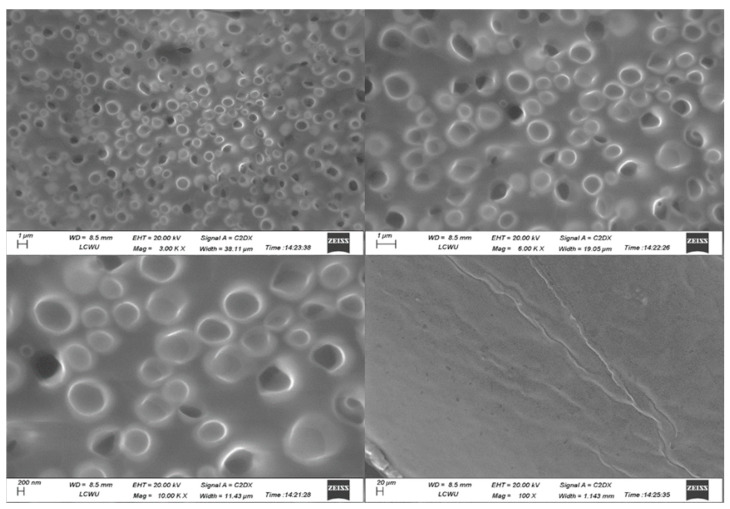
SEM analysis of optimized formulation.

Figure 7 illustrates the examination of the morphology and structure of the formulated product using scanning electron microscopy. The SEM image was taken at 100 X, 10.00 KX, 3.00 KX, and 6.00 KX. Images show the morphology and structure of pure drugs, poloxamers, and the prepared formulations. This image highlighted the fine and homogenous globular structure of nanocarriers, which indicates the proper encapsulation of terbinafine HCl in the nanocarriers.

### 2.10. Particle Size Analysis

**Figure 8:**
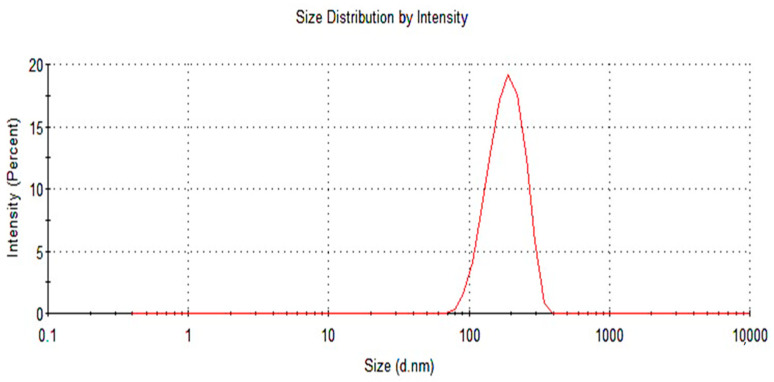
Particle size analysis of an optimized formulation.

Particle size is a crucial parameter that is of very high importance while formulating a nanoemulsion. Initially, the formulation was thinned out, and the particle size was evaluated using the Dynamic Light Scattering (DLS) technique at a temperature of 25 °C [25]. Figure 8 demonstrates that the particle size of the optimized formulation was 272.2 nm.

### 2.11. Zeta Potential

**Figure 9:**
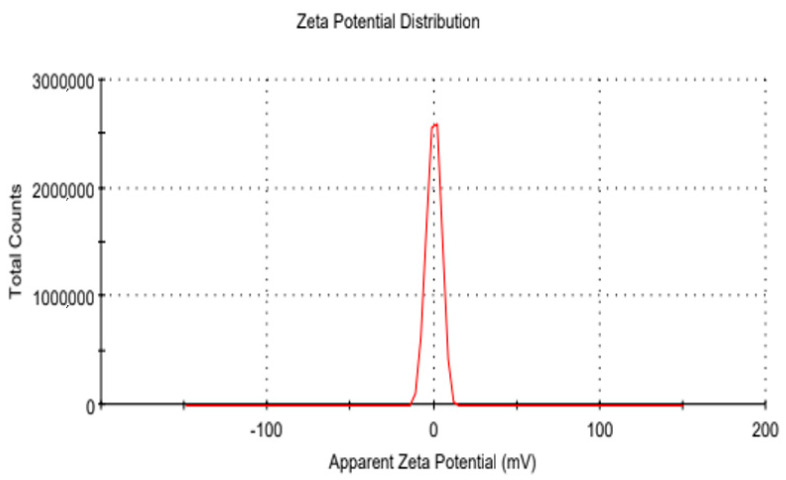
Zeta potential of optimized formulation.

The stability, solubility, and clearance of the components within the formulation are contingent upon the surface charge they exhibit, as shown in Figure 9. The Zeta potential (measured in millivolts, mV) was determined using the Laser Doppler Velocimetry (LDV) technique. All the measurements were conducted in triplicate. For the measurements, samples were diluted 10-fold with water, and measurements were performed in disposable cuvettes at a temperature range of 32–40 °C [26].

### 2.12. In Vitro Drug Release Studies

**Figure 10:**
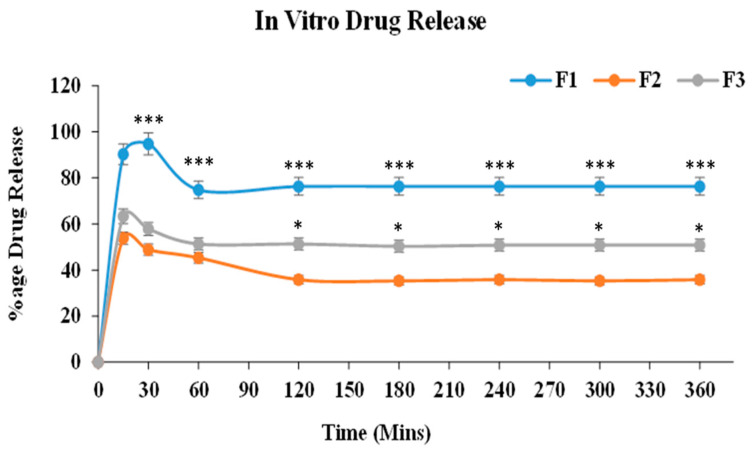
In vitro drug release studies of optimized formulations. For all graphs, each curve represents an individual group, and error bars are expressed as means ± SEM. (* *p* < 0.05; *** *p* < 0.001).

A buffer of pH 7.4 was used as a dissolution medium, and cellophane membrane has been used for the drug release studies. At intervals of 1–24 h, the percentage of drug release was observed. The samples F1, F2, and F3 were extracted after 15, 30, 60, 120, 180, 240, 300, and 360 min, as well as evaluated by a UV-spectrophotometer at 274 wavelengths. Sample absorbance was noted. Figure 8 shows the percentage drug release of the formulations. The drug release of prepared formulations as F1, F2, and F3 carried out in 7.4 pH PBS indicated initial burst release of 90.3%, 53.6%, and 63.3%, respectively, in 15 min. In 30 min, the maximum drug release in the F1 formulation was 94%, and the drug release profile remained constant after 45 min. F1 showed a maximum drug release of 90% as compared to F2 and F3. The drug release percentage is in accordance with time (min) and is shown in a graphical representation. The drug release ability of terbinafine HCl from the formulation was calculated by making dilutions in buffer with a pH of 7.4, as shown in Figure 10.

### 2.13. Drug Permeation Studies

**Figure 11:**
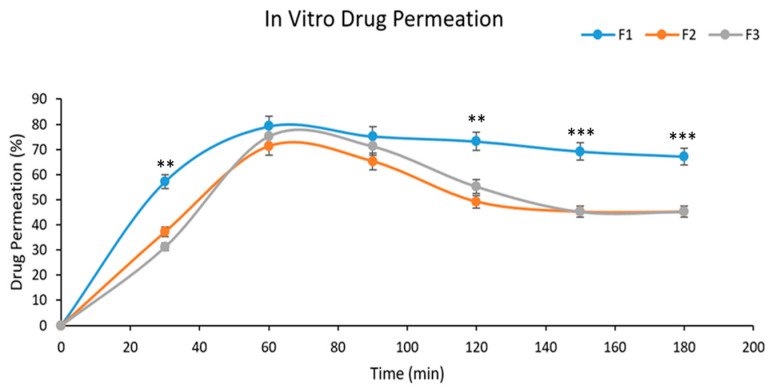
Permeation studies of the optimized formulation. For all graphs, each curve represents an individual group, and error bars are expressed as means ± SEM. (** *p* < 0.01; *** *p* < 0.001).

Drug permeation investigations were conducted within a buffer solution with a pH of 7.4. At some intervals between 1 and 24 h, samples were taken after different intervals of time from the start of the process, and at 274 nm wavelengths, the absorbance was recorded. The graphical representation of the percentage drug permeation of the formulation through the membrane In Figure 11, Formulation F1 was shown to have good drug permeation in comparison to F2 and F3, due to the suitable amount of poloxamers used in the formulation. The percentage of drug permeation from formulation F1 was found to be 79.2% in 60 min.

### 2.14. Antimicrobial Activity

**Figure 12:**
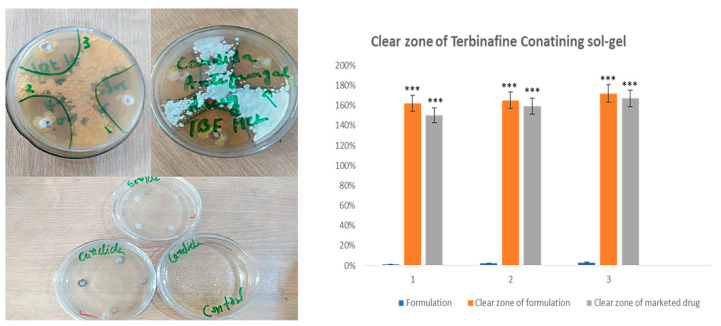
Antimicrobial activity of an optimized formulation. For all graphs, each column represents an individual group, and error bars are expressed as means ± SEM. (*** *p* < 0.001).

Antimicrobial activity was observed in the Petri dishes incubated for 5–7 days. After every 24 h, samples were observed for any changes. During this process, cautions were taken so that no disturbance could affect the antimicrobial activity. A very suitable amount of zone of inhibition was observed in both the prepared formulation and the marketed formulation, indicating that the growth of microorganisms was drastically reduced by the prepared formulation in the time provided, as indicated in Figure 12. The difference between the optimized formulation and the marketed formulation is non-significant but slightly higher, which may be a reason for the increased solubility and permeability of the poloxamer-based formulation.

## 3. Discussion

In this current research, 10 trials of terbinafine-containing sol-gel-based nanocarriers were carried out, out of which the best three (optimized) were selected for further testing, including FTIR, XRD, DSC, viscosity, pH, etc. The prepared formulations also passed the clarity test at room temperature. The pH of the formulation measured was 5.7. As mentioned in the results, the pH of all the formulations lied within the skin pH range (4.2–6.4) [27], signifying their nonirritant nature. The temperature within the solution was precisely assessed by immersing the thermometer into the sample system to ascertain the gelation temperature of the formulation, as indicated in Table 1. The viscosity of the sol-gel-based nanocarriers was measured through a viscometer, which is 15,500 mPa at 60.0 RPM and spindle S3. In comparison, the TBH nanogels had an average viscosity of 37,320 cP, or 725 cP, as determined by a Brookfield viscometer. The control gels’ viscosities were 35,300 cP and 555 cP [28].

The FTIR spectrum of the optimized formulation (F1) (Figure 4d) showed that the characteristic peaks of terbinafine remained unchanged. They were identical to the ideal characteristic peaks of terbinafine (Figure 4a). Peaks for Poloxamer 188 (Figure 4b) and Poloxamer 407 (Figure 4c) were also observed in this spectrum. From the above observations, it was concluded that significant bands of terbinafine and excipients were present in the spectra of the formulation, and they did not show any additional peaks, so there was no chemical interaction between them [29].

The FTIR spectrum of terbinafine (depicted in Figure 4a) was examined within the wavelength range of 500 cm^−1^ to 4000 cm^−1^. FTIR analysis conclusively verified the absence of both chemical and electrostatic interactions among the constituents within the formulated mixture. The lack of any emergent peaks or alterations in functional groups further substantiates the absence of interactions among the individual components.

Furthermore, the sol-gel-based nanocarrier-prepared substance displays consistent and typical bands within its FTIR spectra. On the contrary, the encapsulation of terbinafine within polycaprolactone (PCL) nanoparticles is observed. PCL exhibits distinctive peaks at specific wavenumbers: 1728.09 cm^−1^ for carbonyl stretching, 2939.97 cm^−1^ and 2855.85 cm^−1^ for asymmetric and symmetric CH2 stretching, 1241.29 cm^−1^ for asymmetric COC stretching, and 1186.97 cm^−1^ for OC=O stretching [30]. The carbonyl stretching is also evident in the nanoparticle spectra at 1729.09 cm^−1^ with relatively lower absorbance, considering that the PCL spectra consisted of pure polymers and thus had a higher concentration compared to the nanoparticle solution. Such a kind of interaction was not seen in our formulation, which was an indication of enhanced stability [31].

The DSC thermograms of poloxamer 407, poloxamer 188, and terbinafine were observed, as depicted in Figure 5. The DSC thermogram of the formulation containing terbinafine displayed a distinct endothermic peak, as illustrated in Figure 4d. In the formulation of the DSC thermogram, no new peaks emerged. Additionally, there was no alteration in the shape of the peak or its starting point. These findings suggest that the drug’s chemical integrity remained intact, indicating the absence of any interaction between the drug and the polymer. The XRD pattern of the drug (Figure 6) indicates the peaks of terbinafine that were previously reported [31,32]. The XRD diffractogram of the formulation showed an intense peak, indicating the crystalline nature of the drug. Various particles were observed, well segregated from each other, indicating a stable formulation. Particle size was measured through the test, and it is a crucial step for the formulation. This was measured with a zeta sizer, and the value of the test was found to be 428 nm.

The antifungal agent terbinafine hydrochloride is perfectly suited for addressing infections resulting from dermatophytes. Its high lipophilicity facilitates its accumulation within adipose tissue, skin, and nails, rendering it well-suited for such therapeutic applications [33]. Dynamic light scattering (DLS) was effectively employed for the generation and characterization of nanoparticles. Notably, the nanoparticles demonstrated low polydispersity indices (PDI), and low PDI is an indicator of higher particle stability. As such, it is reasonable to deduce that the nanoparticle solution maintains a monodisperse nature [34]. The zeta potential of sol-gel-based nanocarriers was analyzed, and the zeta potential value of the formulation (Figure 9) was measured and found to be 0.0437, indicating a stable formulation [35].

**Figure 13:**
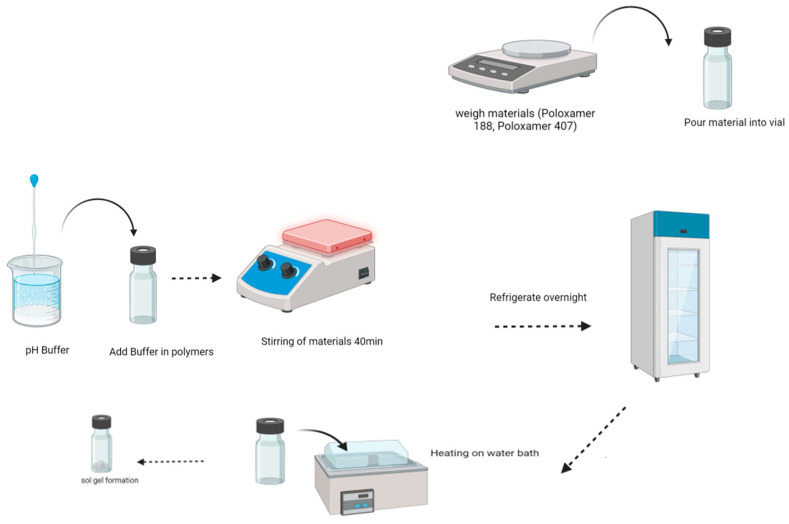
Schematic diagram of the sol-gel-based nanocarriers preparation method.

For conducting in vitro drug release experiments, the USP II dissolution apparatus was employed. As depicted in Figure 10, the formulation exhibited appropriate drug release during the initial phases, transitioning into a sustained release pattern over time with a drug release percentage of 94% [36]. The graph between the percentage of drug permeation and time was plotted. Figure 13 shows the graphical representation of the percentage drug permeation of the formulation through the membrane. The percentage of drug permeation studies was 79.2%. The formulation has shown good drug permeation and enhanced bioavailability due to the suitable amount of poloxamers used in the formulation.

To compare the antifungal effectiveness of sol-gel-based nanocarriers and a commercial product (Lamisil cream) against Candida albicans, the cup-plate diffusion method was used. Candida albicans was clinically isolated, subcultured on SDA medium, and incubated in Petri dishes for five days at 30 °C. Candida albicans was initially discovered as a cultivated organism by closely examining colonial morphology [37]. The results described the effective antifungal activity of terbinafine HCl in sol-gel-based nanocarrier formulations as an indicator of enhanced solubility and permeability, along with higher stability.

## 4. Conclusions

The sol-gel-based nanocarriers approach has now been extensively used to exploit drugs at the target site. In the current research, using the cold method, a stable and suitable sol-gel-based nanocarrier of terbinafine has been developed and evaluated successfully. SEM images have indicated the formation of suitable particles that are well segregated from each other. FTIR has confirmed the presence of functional groups of drugs and excipients in the prepared formulation. Permeation study results confirmed the effective terbinafine permeation, thus providing a prominent anti-fungal effect. The in vitro studies had proven that terbinafine could be a very good candidate for the treatment of onychomycosis. Hence, the prepared formulations can be delivered via transdermal drug delivery as an effective route of administration for rapid drug delivery for treating onychomycosis. Compared to various formulations, sol-gel-based nanocarriers have the advantage of overcoming the multiple disadvantages of gels. Further, the sol-gel-based nanocarriers approach is very appropriate for loading poor water-soluble drugs to improve their bioavailability, enhance the efficacy of drugs at low doses, and avoid re-administration of gel again and again. Such formulations can be considered a better alternative for the commercial application of anti-fungal drugs.

## 5. Material and Methods

### 5.1. Materials

Terbinafine HCl was obtained from the Novartis Research Institute in Vienna, Austria. Polymers such as Poloxamer-188 and Poloxamer-407 were sourced from Sigma-Aldrich in Darmstadt, Germany. The other materials used are of analytical grade.

### 5.2. Method of Preparing Sol-Gel-Based Nanocarriers of Terbinafine HCl

Sol-gel-based nanocarriers were prepared by using the “cold method,” schematically represented in Figure 13 [38,39]. Poloxamers 407 and 188 were mixed in the required quantity of distilled water and stirred on a magnetic stirrer for 4 h at 300 rpm to control the formation. Then, the mixture was kept in refrigeration at 4 °C overnight. Terbinafine was dissolved in 7.4 pH phosphate buffer, added 2 mL to the prepared sol-gel-based nanocarriers, stirred, and again kept in refrigeration for 8 h. In addition, its pH was adjusted, and gelation time was measured. Further, ten formulations were prepared by varying the concentration of poloxamer with the drug, as shown in Table 1.

### 5.3. Characterization and Evaluation

#### 5.3.1. Formulation Optimization

Different trials of the formulation were designed using Design Expert (DoE) software (version 13). The Central Composite Design (CCD) of the Response Surface Methodology (RSM) was employed within the DoE. RSM includes various techniques and mathematical methods necessary in the modeling and analysis of scenarios, whereas the response is affected by multiple variables [40]. The composition of the optimized formulation is mentioned in Table 2. Out of 10 design formulations, the best of three formulations was selected, among which the optimized formulation was F1. In Table 2, several trials have been generated and described as coded levels. To identify the main effect and interaction between poloxamers 188 and 407 at varying concentrations, a central composite design of the response surface methodology has been used. In order to determine the optimal formulation, the gelation temperature of sol-gel-based nanocarriers was used as an indicator. The gelation temperature was measured by fitting the response surface model to 10 formulation runs obtained from the central composite design. The process of optimization was carried out by using different concentrations of both poloxamers using a two-factor and quadratic model design. The formulation was also analyzed statistically using Design Expert software [41]. Table 6 shows the various coded levels of RSM and Table 7 shows the composition of poloxamer in all trials.

After putting the data within the polynomial equation (Equation (3)), an analysis of variance (ANOVA) was carried out employing a 95% confidence interval [41].

Y = β_0_ + b1X_1_ + b_2_X_2_ + b_12_X_1_X_2_ + b_1_X_1_^2^ + b_2_X_2_^2^ (3)

The arithmetic mean response of running trials is β0, and the dependent variable is represented by Y. In this study, X_1_ and X_2_ are two variables that were changed from low to high values. A positive or negative result is judged by a polynomial equation.

#### 5.3.2. Clarity

The clarity of sol-gel-based nanocarriers was determined by visual analysis [42].

#### 5.3.3. pH

The ADWA pH meter was used to determine the pH of the prepared formulation. The pH of skin ranges from 5–6.5, and it is necessary that the prepared formulation be within this range [42]. The pH of the prepared formulation was analyzed by dipping the electrode of a pH meter in the solution.

#### 5.3.4. Viscosity

Viscosity is a crucial characteristic of topical formulations. It has a significant impact on the drug release profile. Additionally, the viscosity of semisolid dosage forms influences their stability, spread ability, and application to the skin [42]. To determine viscosity, 20 mL of sol gel was filled in a glass vial and subjected to the viscometer, assembled with spindle S3. The viscosity of in-situ gel should be up to 16,000 cps [43].

#### 5.3.5. Gelation Temperature

The gelation temperature of sol-gel-based nanocarriers was determined by using the magnetic stirring method [44]. First, 5 mL of each diluted sol-gel-based nanocarrier solution was placed in clear glass vials and kept at a temperature of 4 °C. A magnetic stir bar was placed in each sol-gel-based nanocarrier sample after a 2 h gap and then increased the temperature gradually at a rate of 1 °C per minute. A thermometer was inserted into the sol-gel-based nanocarrier mixture to monitor the gelation temperature. The temperature at which the magnetic bar stopped stirring and the sol-gel-based nanocarrier material appeared to be non-flowing was considered the gelation temperature [45].

#### 5.3.6. Fourier Transform Infrared Spectrophotometer (FTIR)

FTIR is an analytical procedure for the evaluation of a chemical compound. FTIR analysis required a small quantity of all materials as well as formulation. Data on infrared transmittance were analyzed at ranges of 600 to 3800 cm^−1^. Spectrum was recorded on a FTIR instrument (Perkin Elmer Spectrum Two, Waltham, MA, USA), and the data were processed by means of a PC-based software-controlled instrument [46].

#### 5.3.7. Thermal Stability Analysis (DSC/TGA)

The thermal stability of the optimized formulation was analyzed by a differential scanning calorimeter (DSC) and thermogravimetric (TG) analyzer (PerkinElmer STA 6000, Inc. 940 Winter Street Waltham, MA 02451, USA). For DSC analysis, a portion of the formulation was placed within sealed aluminum pans. The sample was then subjected to scanning from 25 to 400 °C at a heating rate of 10 °C per minute. From the DSC thermograms of the samples, a peak transition temperature was observed [47].

#### 5.3.8. X-ray Diffraction (XRD)

An X-ray diffraction (XRD) analysis is used to characterize the structural arrangement of crystalline materials on a long-range scale and the arrangement of non-crystalline materials on a short-range scale [47]. With the use of an X-ray diffractometer (JDX-3523, Tokyo, Japan), an X-ray diffraction investigation of prepared sol-gel-based nanocarriers and pure drugs was performed. Both the samples have been detected between 5° and 60° using 2θ at a rate of 3°/min [48].

#### 5.3.9. Scanning Electron Microscopy (SEM)

Surface microbiology and particle size analysis were observed by scanning electron microscopy (SEM) [34]. SEM (EVO LS10 Zeiss, Jena, Germany) is implemented as a major technique to study the three-dimensional structure of the sol-gel-based nanocarriers. The prepared sample was applied to the grid and dried. Then the sample was taken and analyzed under different magnifications [49].

#### 5.3.10. Particle size Analysis

The particle size of the prepared sol-gel-based nanocarriers was measured using a Malvern Zetasizer (manufactured by Malvern Instruments, Malvern, UK). Initially, the prepared formulation was diluted with distilled water, then placed in a cuvette and analyzed for particle size determination [49].

#### 5.3.11. Zeta Potential

The analysis of zeta potential was performed using the Malvern Zetasizer (Malvern Instruments, Malvern, UK). Every particle has a surface charge that is known as zeta potential. The normal range of zeta potential is +30 to −30 mV [37]. It was evaluated by implementing Lasee Doppler Velocimetry (LDV) mode [50].

#### 5.3.12. In Vitro Drug Release Studies

A USP II dissolution apparatus was employed to conduct an in vitro drug release study. Sol-gel-based nanocarriers of terbinafine HCl were subjected to in vitro drug release testing in 500 mL of a phosphate buffer (pH 7.4) at 37 °C. A dialysis membrane of pore size (M.W.C.O. 12,000–14,000) containing 2 g of the formulation was attached to the paddle by a thread at 50 rpm. Approximately a 2 mL sample was collected through a pipette at 15 min, 30 min, 60 min, 120 min, 180 min, 240 min, 300 min, and 360 min intervals. Meanwhile, an equal volume of phosphate buffer (pH 7.4) was added as a dissolution medium. The concentration of terbinafine HCL was further quantified by the UV method [51].

#### 5.3.13. Drug Permeation Studies

In vitro drug permeation studies were conducted using a Franz diffusion cell (Variomag Telesystem, H+P Labortechnik, Oberschleißheim, Germany). This is a simple and direct method for accurately assessing the in-vitro release of drugs from topical formulations like creams, ointments, liposomal formulations, and gels. By using this technique, the interconnection between skin, drug, and formulation characteristics can be easily analyzed [50]. The donor compartment contains a 1 mL sample, and the recipient compartment contains 7 mL of phosphate buffer with a pH of 7.4 [51]. The drug permeation was measured by diluting the sample with receptor medium and analyzed by a UV spectrophotometer set at 274 nm [52].

#### 5.3.14. Antimicrobial activity

The antimicrobial activity of formulated terbinafine sol-gel-based nanocarriers against *Candida albicans* was evaluated using the “cup plate technique” on Sabouraud dextrose agar. A pure microbial culture was prepared and incubated with a solution of Sol-gel-based nanocarrier formulation, a marketed drug, and the pure drug at a concentration of 1 mg/mL. Plates, along with the control, were incubated at 27 °C for 48 h, after a 2-h gap. The formation of clear zones of inhibition around each cup was observed, and these inhibition zones were subsequently compared to each other [53].

#### 5.3.15. Statistical Analysis

All the collected results were expressed as the mean ± standard deviation after analysis by one-way ANOVA. For all graphs, each column represents an individual group, and error bars are expressed as means ± SEM. (* *p* < 0.05; ** *p* < 0.01; *** *p* < 0.001).

## Figures and Tables

**Table 1:** Experimental design of independent variable parameters.

Run	(X1) Polox407 (%)	(X2) Polox88 (%)	(R1) Gelation Temp (°C)	(R1) Gelation Time (_S_)
1	10	15	32.8	0.702513
2	10	12.5	35.9	0.910204
3	20	10	30	0.59545
4	10	12.5	35.9	0.869
5	15	10	37	0.95282
6	20	12.5	34	0.80619
7	15	15	38.2	1.095
8	15	15	38.2	1.095
9	20	15	32	0.70619
10	10	10	40.1	1.19751

**Table 2:** ANOVA for gelation temperature and gelling time.

Variables	Gelation Temperature °C	Gelling Time (s)
F1	38.82	67
A (poloxamer 407(A_1_))	−1.84	−0.09
B (poloxamer 188 (B_2_))	−0.36	−0.0125
AB	2.33	0.1514
A2	−3.87	−0.22
B2	−1.22	−0.047
Model *p* value	0.01	0.001
R^2^	0.85	0.77
Adjusted R^2^	0.73	0.58
F Value	7.09	4.16

**Table 3:** pH of the F1–F3 formulation.

Formulation	Drug (mg)	Poloxamer 407 (mg)	Poloxamer 188 (mg)	pH
F1	10	20	18	5.7 ± 0.18
F2	10	21	13	5.8 ± 0.16
F3	10	25	15	5.9 ± 0.18

**Table 4:** Viscosity of the prepared formulation.

Formulation	Viscosity (Cps)
F1	15500 ± 2.45
F2	12900 ± 3.26
F3	16000 ± 2.98

**Table 5:** Gelation temperature of the prepared formulation.

Formulation	Gelation Temperature (°C)
F1	28 ± 0.09
F2	34 ± 0.06
F3	27 ± 0.08

**Table 6:** Coded level of RSM.

Name	Units	Low	High	−Alpha	+Alpha
Polox 407	%	10	20	4.26	5.72
Polox 188	%	10	15	2.13	4.84

**Table 7:** Composition of the trial formulation.

Trials	Drug (mg)	Poloxamer 407 (mg)	Poloxamer 188 (mg)
1	10	18	15
2	10	21.5	17.5
3	10	25	15
4	10	18	20
5	10	21.5	13.96
6	10	21	13
7	10	16.55	17.5
8	10	26.5	12.5
9	10	20	18
10	10	25	30

## Data Availability

All the data are contained in the manuscript.
